# Use of Population-based Surveillance to Define the High Incidence of Shigellosis in an Urban Slum in Nairobi, Kenya

**DOI:** 10.1371/journal.pone.0058437

**Published:** 2013-03-07

**Authors:** Henry N. Njuguna, Leonard Cosmas, John Williamson, Dhillon Nyachieo, Beatrice Olack, John B. Ochieng, Newton Wamola, Joseph O. Oundo, Daniel R. Feikin, Eric D. Mintz, Robert F. Breiman

**Affiliations:** 1 Global Disease Detection Program, Kenya Medical Research Institute (KEMRI)-Centers for Disease Control and Prevention-Kenya (CDC-K) Collaboration, Nairobi and Kisumu, Kenya; 2 National Center for Emerging and Zoonotic Infectious Diseases, Centers for Disease Control and Prevention, Atlanta, Georgia, United States of America; The Australian National University, Australia

## Abstract

**Background:**

Worldwide, *Shigella* causes an estimated 160 million infections and >1 million deaths annually. However, limited incidence data are available from African urban slums. We investigated the epidemiology of shigellosis and drug susceptibility patterns within a densely populated urban settlement in Nairobi, Kenya through population-based surveillance.

**Methods:**

Surveillance participants were interviewed in their homes every 2 weeks by community interviewers. Participants also had free access to a designated study clinic in the surveillance area where stool specimens were collected from patients with diarrhea (≥3 loose stools within 24 hours) or dysentery (≥1 stool with visible blood during previous 24 hours). We adjusted crude incidence rates for participants meeting stool collection criteria at household visits who reported visiting another clinic.

**Results:**

*Shigella* species were isolated from 224 (23%) of 976 stool specimens. The overall adjusted incidence rate was 408/100,000 person years of observation (PYO) with highest rates among adults 34–49 years old (1,575/100,000 PYO). Isolates were: *Shigella flexneri* (64%), *S. dysenteriae* (11%), *S. sonnei* (9%), and *S. boydii* (5%). Over 90% of all *Shigella* isolates were resistant to trimethoprim-sulfamethoxazole and sulfisoxazole. Additional resistance included nalidixic acid (3%), ciprofloxacin (1%) and ceftriaxone (1%).

**Conclusion:**

More than 1 of every 200 persons experience shigellosis each year in this Kenyan urban slum, yielding rates similar to those in some Asian countries. Provision of safe drinking water, improved sanitation, and hygiene in urban slums are needed to reduce disease burden, in addition to development of effective *Shigella* vaccines.

## Introduction

Although rates have decreased in middle-income and wealthy nations, shigellosis remains a major public health problem in impoverished settings because of sub-optimal sanitation and hygiene and contaminated water supplies [1], [2], [3]. Annually, *Shigella* is responsible for >160 million infections in developing countries with an estimated 1.1 million deaths [3]. Most deaths occur in children <5 years of age [3]. Humans are the only natural hosts of *Shigella*. Persons with diarrhea are primarily responsible for transmission [4], which occurs predominantly through the faecal-oral route via direct contact, fomites, or contaminated food and water [5]. The low infectious dose (as few as 10 organisms) renders *Shigella* highly infectious [5]. When human feces are not properly disposed of, common houseflies, particularly *Musca domestica*, may serve as vectors for transmission [6].

Symptoms of shigellosis typically include diarrhea and/or bloody stools with abdominal cramps and tenesmus [7]. Antimicrobial drugs shorten duration of illness and bacterial shedding, and decrease morbidity and mortality [8]. Antimicrobial resistance among *Shigellae* is increasing worldwide [9], [10], [11], [12]. In sub-Saharan Africa, repeated prolonged outbreaks of drug-resistant *Shigella* have reduced antimicrobial therapeutic options and increased cost of therapy [13].

Previous estimates of the burden of shigellosis in developing countries may have underestimated its magnitude. Studies present varying incidence and prevalence rates, because of divergent methods and intrinsic disparities among the populations studied [2], [14], [15], [16]. Rapid urbanization in Africa and Asia has increased the number of residents of informal settlements who lack access to basic sanitation and safe drinking water. Continued urbanization in Africa will likely contribute to an even greater burden of enteric diseases including shigellosis.

Since 2005, the Centers for Disease Control and Prevention (CDC) and the Kenya Medical Research Institute (KEMRI) have maintained population-based infectious disease surveillance (PBIDS) in a geographically defined area within an urban informal settlement in Nairobi, Kenya [17], [18]. We used systematically collected data from PBIDS to define the incidence and characterise the epidemiologic, clinical, and microbiologic features of shigellosis within this population.

## Methods

### Study site

The urban PBIDS system is based in Kibera, a large informal settlement (urban slum) in Nairobi, Kenya. Kibera lacks adequate sanitation facilities; human and animal wastes drain into open sewage runoff. Drinking water is often obtained from unregulated vendors using illegal connections to a municipal piped water system, a situation that facilitates contamination of water supplies. Food is traded at unregulated outdoor markets that often lack basic hygiene standards.

### Surveillance procedures

The PBIDS procedures have been described previously [17], [19]. Briefly, we conducted population-based surveillance for the etiologies of diarrheal disease, febrile illness, pneumonia and jaundice within two of 12 villages in Kibera: Gatwikera and west Soweto. The average population of the surveillance area is 28,500 persons in a 0.37 km^2^ area (population density = 77,000 persons per km^2^). The surveillance area was divided into 10 pre-defined geographic zones roughly based on pre-existing sub-village designations. Trained community interviewers visited enrolled participants fortnightly from March 1, 2007 through Jan 31, 2010 and, thereafter every week collecting data on illnesses and deaths since the previous visit. The frequency of household visits was increased to shorten the recall period during the pandemic of influenza A (H1N1). Shortened recall period was found to improve accuracy of data collected during household visits [19]. Community interviewers also encouraged participants to access, whenever they fell ill, a free well-staffed and equipped study clinic (Tabitha clinic) owned by Carolina for Kibera and located within 1 km of all surveillance area residences.

### Stool collection criteria

From January 1, 2007 through December 31, 2010, we collected stool samples from consenting patients who met the following criteria:

1. Uncomplicated diarrhea (defined as ≥3 loose stool within a 24-hour period) without signs or symptoms of dehydration or dysentery. (To minimize burden on the laboratory we sampled a maximum of 6 stool specimens from patients with uncomplicated diarrhea per day: 3 from patients <5 years old and 3 from patients ≥5 years old);
2. Complicated diarrhea based on the presence of symptoms or signs of dehydration, defined as: drinking eagerly or unable to drink or breastfeed, vomiting everything, slow skin pinch return (≥2 seconds), irritability, sunken eyes, lethargy or unconsciousness (all patients);
3. Dysentery, defined as reported or visible blood in ≥1 stool within 24 hours of clinic visit (all patients).

A case of shigellosis was defined as a patient meeting any of the above criteria who had *Shigella* isolated from stool.

### Laboratory procedures

We provided each consenting participant meeting the stool collection criteria with a labelled stool container and instructions on how to sample their stool; placing emphasis on including blood or mucus, if present. From May 2008, patients visiting the clinic who were unable to provide a stool sample while at the clinic were sent home with a labelled container to collect a stool specimen. A sample collector was then sent to the patient's home to collect the specimen within 4 hours after the clinic visit. Swabs of whole stool specimens were placed in Cary-Blair transport medium, refrigerated at 4–8 °C then transported to the KEMRI-CDC enteric microbiology laboratory in Kisumu from Jan 2007 to May 2009; thereafter, stool specimens were processed in a microbiology laboratory at the study clinic. More than 80% of specimens were processed within 24–28 hours after collection. Specimens were processed using standard microbiological procedures [20]. Isolates were tested using the Kirby-Bauer disc diffusion technique [21] for susceptibility to ampicillin, amoxicillin/clavulanic acid acid, ceftriaxone, chloramphenical, nalidixic acid, ciprofloxacin, gentamycin, kanamycin, streptomycin, sulfisoxazole, tetracycline, and trimethoprim-sulfamethoxazole. Results were interpreted according to Clinical Laboratory Standards Institute (CLSI) guidelines [22]. For quality control, we used Escherichia coli ATCC 25922 for growth and antimicrobial testing monitoring. We also participated in external quality assurance protocols as provided by the World Health Organization (WHO) and the South African National Institute for Communicable Diseases (NICD).

### Analysis

Data were analysed using SAS version 9.1 (Cary, NC). We calculated incidence rates as the number of shigellosis cases per 100,000 person-years of observation (PYO), as described previously [18], [23]. Participants included all individuals living in consenting households within the surveillance area for a minimum of 4 months between 1 January 2007 and 31 December 2010. Person-years of observation were calculated by totalling person-days for all people who met the residence requirement during each biweekly home visit and dividing the total number of person-days by 365.25.

In calculating crude incidence rates, we divided the number of *Shigella* cases by the PYO. Our fortnightly and weekly home visit data, provided real time health utilization data for use in assessing what proportion of shigellosis cases were likely missed through clinic-based diarrheal illness testing. Thus, adjusted rate calculations were based on the number of patients who met each of the three stool sample collection criteria (from data collected during home visits) who visited clinics other than the study clinic for their illness; thus, they were ill enough to visit a clinic for their diarrhea or dysentery, but no specimens would have been obtained, as specimens were only collected and processed from patients attending the study clinic. Similar incidence adjustment methods based on health utilization data have been used in other recent studies [18], [24]. Thus, we divided number of residents meeting each of the stool collection criteria on home visit who visited the study clinic by number of residents meeting each of the same criteria who visited any clinic for that illness. Episodes of diarrhea/dysentery not associated with visits to any clinic were not “counted” in the adjustment. This proportion was then divided into the crude rate of shigella obtained for each criterion.

**Figure 1:**
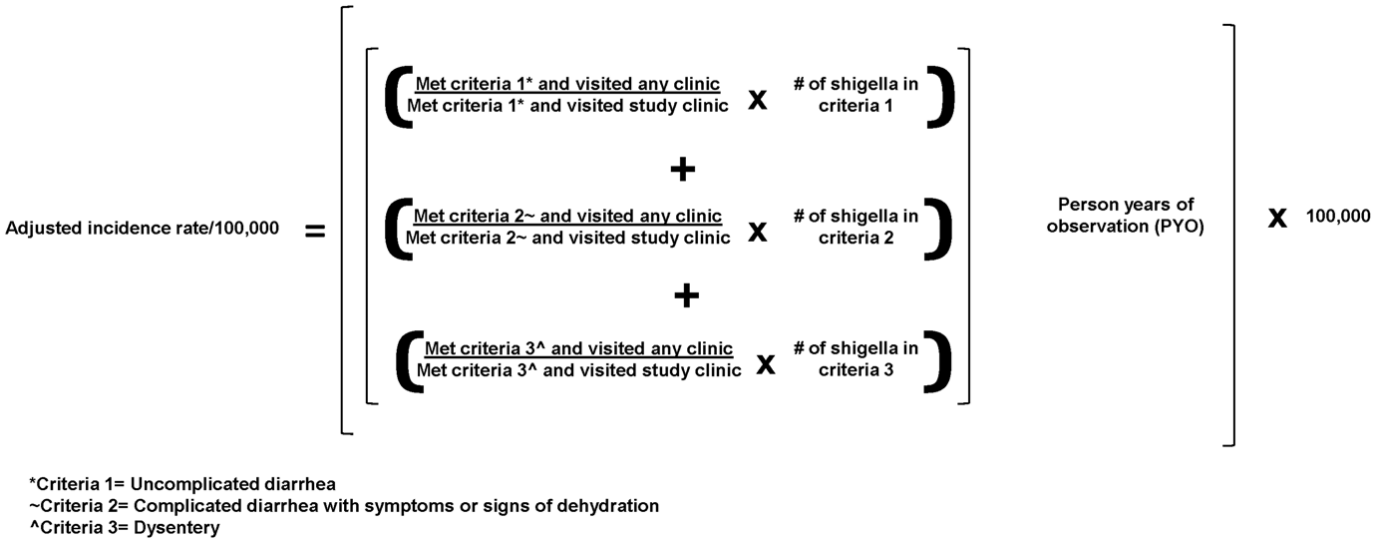
Formula for adjusted incidence rate calculations.

The overall adjusted incidence rate was calculated by summing the adjusted *Shigella* numbers for each stool collection criterion and dividing this sum by the total number of person years contributed by participants in the study population (Fig. 1). These calculations were also done by age group, gender, and by geographic zone.

For characterizing clinical features, drug susceptibility and *Shigellae* species distribution, we included data for the entire study period (1 Jan, 2007 to 31 Dec, 2010). From January, 2007, through April, 2008, few (<5%) patients who met stool collection criteria provided a stool sample for testing, leading to imprecise adjusted incidence rates. Thus, we excluded data from that time period for calculating the crude and adjusted incidence rates. From May 2008, a sample collector was engaged to collect stool samples from patients' homes when patients were unable to provide a sample at the clinic. This resulted in a fivefold increase in the proportion of patients meeting case definitions who provided a stool sample for stool culture, and provided more stable numbers for rate calculations.

Logistic regression analysis was used to determine association of clinical features and shigellosis for patients seen at the clinic. Significant unadjusted clinical features (p<0.05) were then entered into a multivariate model to control for confounding. We also examined household data collected from patients confirmed to have shigellosis for a period of one month after the clinic visit when the diagnosis was made to assess the number of hospitalizations and deaths

We used incidence rates from this study to project the burden of shigellosis among the estimated 4.3 million people living in informal settlement areas in Kenya [25]. We estimated the age structure of persons living in informal settlements to be similar to that in Kibera. Monthly rainfall data for each of the three years was obtained from the National Oceanic and Atmospheric Administration (NOAA) website specifically for Nairobi region [26] and were used to explore possible associations with *Shigella* incidence over time using Pearson correlation coefficient. P-values <0.05 were considered significant for all statistical tests.

### Ethical review

The protocol, surveillance questionnaires and consent forms were reviewed and approved by the Ethical Review Committee at the Kenya Medical Research Institute (protocol number 1899) and the Institutional Review Board of CDC- Atlanta (protocol number 4566).

## Results

### Incidence rate calculations

#### Age and sex distribution of surveillance participants

Between 1 May, 2008, and 31 Dec, 2010, 77,939 PYO were contributed by persons enrolled into the surveillance system. Adults 18–34 years of age accounted for 35% of the population under surveillance, followed by young adults 10–17 years who accounted for 16%. Males and females contributed equally. The highest proportion of surveillance participants were from Zone 1; 18% followed by zone 10; 16% (Table 1).

**Table 1:** Person years of observation, crude and adjusted incidence of *Shigella* by age category, sex, zone and year, 1 May 2008 to 31 Dec 2010, Kibera, Kenya.

	Person years of observation (PYO)	Incidence rates	
		Crude incidence/100,000	Adjusted incidence/100,000
Age category			
<12 m	2,369 (3)	84	136
12–**23** **m**	2,954 (4)	203	273
24–59 m	8,936 (11)	291	369
5–9 y	12,070 (15)	141	175
10–17 y	12,765 (16)	212	268
18–34 y	26,968 (35)	356	559
35–49 y	4,207 (5)	1046	1575
50 y+	7,669 (10)	78	108
Sex			
Males	39,090 (50)	219	306
Females	38,849 (50)	356	484
Year			
2008	17,658 (23)	306	392
2009	28,892 (37)	270	363
2010	31,388 (40)	293	428
*Zone			
1	13,741 (18)	393	
2	9,398 (12)	766	
3	2,467 (3)	446	
4	2,828 (4)	601	
5	7,839 (10)	370	
6	8,852 (11)	215	
7	5,111 (7)	274	
8	10,219 (13)	391	
9	5,379 (7)	316	
10	12,105 (16)	256	
Overall	77,939	287	408

* Adjusted incidence rates by zone not shown

#### Household interviews, clinic utilization and case detection

**Figure 2:**
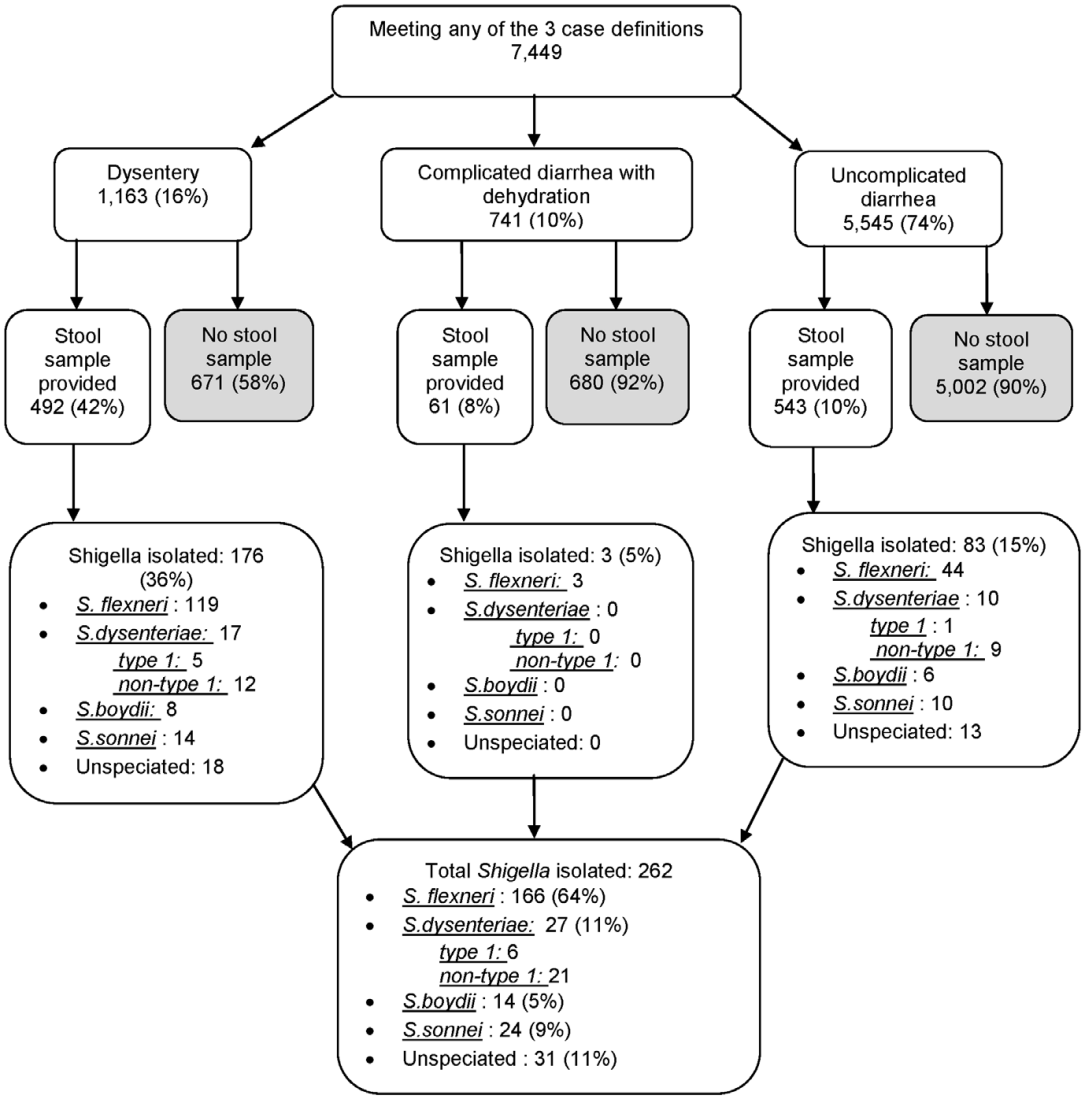
Flow chart illustrating distribution of diarrhea cases and shigella species isolated between 1 Jan 2007 and 31 Dec 2010 in Kibera, Kenya.

**Figure 3:**
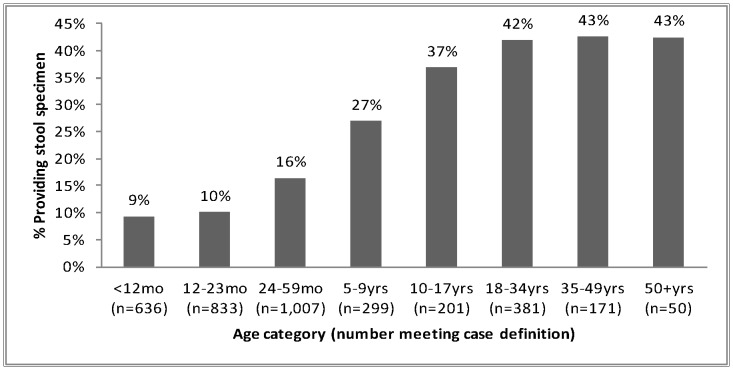
Proportions of patients meeting the case definitions providing stool sample by age category.

Over 80% of enrolled study participants had successful interviews during the biweekly/weekly household visits. By year, this was distributed as follows; 78% in 2007, 74% in 2008, 83% in 2009 and 84% in 2010. During these visits, over 60% of participants who met any of the stool collection criteria and reported visiting any clinic, had visited Tabitha clinic (data not shown). At Tabitha clinic, 7,449 patients met one of the three stool collection criteria (Fig. 2). The majority, 5,545 (74%) had uncomplicated diarrhea, 1,163 (16%) had dysentery and 741 (10%) had complicated diarrhea with dehydration (Fig. 2). Almost half (42%) of those who had dysentery provided a stool sample while only 543 (10%) and 61 (8%) of those who had uncomplicated diarrhea and complicated diarrhea with dehydration provided a stool sample, respectively (Fig. 2). The proportion of patients meeting any of the stool collection criteria who provided a stool sample increased gradually with age, stabilizing at the 18–34 year age group (Fig. 3).

#### *Shigella* isolation and incidence rates

We isolated 262(24%) *Shigella* bacteria from 1,096 stool specimens collected from patients meeting any of the three stool collection criteria from 1 Jan, 2007, through 31 Dec, 2010: 176 (67%) of *Shigella* isolates were from patients presenting with dysentery, 83 (32%) from patients with uncomplicated diarrhea and 3 (1%) from patients with complicated diarrhea with dehydration (Fig. 2). For the period; 1 May, 2008 through 31 Dec, 2010, 242 (23%) *Shigella* bacteria were isolated from 976 stool specimens (data not shown)

The overall crude incidence rate of shigellosis was 287/100,000 PYO (Table 1). Crude incidence rate was highest among adults 35–49 years old (1,046/100,000 PYO). The overall adjusted incidence rate was 408/100,000 PYO with highest adjusted rates in adults 35–49 years old (1575/100,000) (Table 1). Infants and adults 50 years old and above had the lowest adjusted incidence rates (136/100,000 and 108/100,000 respectively). Females were more likely than males to have shigellosis, (OR: 1.52, 95% CI: 1.15–2.02). There was significant variation in crude incidence rates by geographic location, with the rates ranging from 215/100,000 PYO (zone 6) to 766/100000 PYO (zone 2) (p-value <0.001) (Table 1).

### Epidemiology and laboratory characteristics of shigellosis

#### Species distribution

Most *Shigella* isolates were *S. flexneri* (64%) followed by *S. dysenteriae* (11%) *S. sonnei* (9%), and *S. boydii* (5%). Species could not be determined for 12% of isolates; 6 (22%) of the 27 isolates of S. *dysenteriae* were type 1 (Fig. 2).

#### Clinical features for shigellosis

Patients with dysentery were more likely to have *Shigella* isolated from stool than patients with complicated and uncomplicated diarrhea (OR: 10.77, 95% CI: 3.33–34.87) and patients with “uncomplicated” diarrhea were more likely to have evidence of shigellosis than those with a complicated diarrheal illness (OR: 3.49, 95% CI: 1.07–11.39). Patients with history of vomiting (OR: 0.54, 95% CI: 0.33–0.86) were less likely to have shigellosis (Table 2).

**Table 2:** Demographic and clinical characteristics associated with shigellosis on bivariate and multivariate analysis among patients seen in study clinic, 1^st^ Jan 2007 to 31^st^ Dec 2011, Kibera.

			Bivariate analysis		Multivariate analysis	
Characteristic	N	Shigella positive (%)	OR	CI	OR	CI
Age category*						
<12 m	72	2 (3)	Ref			
12–23 m	101	7 (7)	2.13	0.52–12.93	2.36	0.47–11.85
24–59 m	216	34 (16)	4.85	1.53–27.94	5.47	1.26–23.68
5–9 y	117	20 (17)	5.47	1.63–31.88	6.10	1.36–27.38
10–17 y	122	29 (24)	8.16	2.52–47.28	9.34	2.12–41.14
18–34 y	297	115 (39)	16.08	5.32–91.94	17.51	4.15–73.89
35–49 y	136	49 (36)	14.57	4.63–83.91	15.84	3.66–68.55
50 y+	35	6 (17)	6.12	1.38–38.00	6.64	1.24–35.70
Sex*						
Male	526	105 (20)	Ref		Ref	
Female	570	157 (28)	1.52	1.15–2.02	1.47	1.68–2.00
Vomiting**						
No	532	126 (24)	Ref			
Yes	168	24 (14)	0.54	0.33–0.86	—	—
Reported fever						
No	406	87 (21)	Ref			
Yes	311	65 (21)	0.97	0.67–1.39	—	—
Duration of diarrhea						
Less than 3 days	594	156 (26)	Ref			
3–5 days	398	86 (22)	0.77	0.58–1.05	—	—
6–9 days	69	11 (16)	0.53	0.27–1.04	—	—
10 days and more	22	4 (18)	0.62	0.21–1.87	—	—
Belly pain						
No	132	36 (27)	Ref			
Yes	542	175 (32)	1.27	0.83–1.94	—	—
Headache						
No	265	79 (30)	Ref			
Yes	154	43 (28)	0.91	0.59–1.42	—	—
Muscle pain						
No	378	104 (28)	Ref			
Yes	35	13 (37)	1.56	0.76–3.20	—	—
Case presentation*						
Complicated diarrhea with dehydration	61	3 (5)	Ref		Ref	
Uncomplicated diarrhea	543	83 (15)	3.49	1.07–11.39	1.73	0.51–5.85
Dysentery	492	176 (36)	10.77	3.33–34.87	5.32	1.59–17.86
HIV status						
Negative	313	105 (34)	Ref			
Positive	100	33 (33)	0.98	0.60–1.57	—	—
**Totals**	**1,096**	**262**				

* Significant variables on bivariate analysis included into multivariate model

** Significant characteristics on bivariate analysis but not significant on multivariate model hence excluded from final multivariate model

One patient was hospitalized within 2 days of being seen at the clinic while one patient died within 3 weeks after being diagnosed with shigellosis at the clinic. *S. flexneri* was isolated from both patients.

#### Drug susceptibility

Over 80% of *Shigella* isolates were resistant to each of the following drugs: streptomycin, sulfisoxazole, tetracycline and trimethoprim-sulfamethoxazole (Table 3). Three (1%) isolates were resistant to ceftriaxone. Seven (3%) isolates were resistant to nalidixic acid, of which 2 (29%) were also resistant to ciprofloxacin (Table 3). *Shigella* incidence and monthly rainfall Shigellosis cases occurred throughout the year. There were no significant association between *Shigella* incidence and monthly rainfall (Pearson correlation coefficient  = 0.002, p-value = 0.99).

**Table 3:** Drug susceptibility patterns for *Shigella* isolated, Kibera, Kenya.

Antibiotic class	Antibiotic tested	Number of *Shigellae* resistant (%) `N = 222
Penicillins	Ampicillin	147 (67)
	Amoxicillin/clavulanic acid*	69 (35)
Cephalosporin	Ceftriaxzone	3 (1)
Quinolone	Nalidixic acid	7 (3)
Fluoroquinolone	Ciprofloxacin	2 (1)
Chloramphenicol	Chloramphenicol	91 (41)
Aminoglycosides	Gentamycin	5 (2)
	Streptomycin	192 (86)
Sulfonamide	Sulfisoxazole	212 (96)
Sulphonamide and trimethoprime	Trimethoprim-sulfamethoxazole	212 (95)
Tetracycline	Tetracycline	184 (83)

* low denominator due to stock out of Amoxicillin/clavulanic acid disks

`40 isolates did not have drug susceptibility tests done as antibiotics were not available when they were being tested

## Discussion

Our study revealed a high burden of shigellosis within a densely populated urban slum in Kenya. The high crude incidence of shigellosis of 0.29% was slightly higher than that reported in a multicenter population-based study done in six developing countries in Asia; three of the sites were rural/semi rural (in China, Thailand, and Vietnam) and three of the sites were in urban slums (in Bangladesh, Indonesia, and Pakistan), where the overall unadjusted incidence of *Shigella* in this multicenter study was estimated at 0.2% among patients of all ages [27]. The rates were much higher than that seen in industrialized countries such as the UK and US where the incidence of shigellosis is estimated at 0.027% and 0.0038% respectively [28], [29].

In our study, over 20% of all stool samples yielded *Shigella*, further confirming the high burden of shigellosis. Similar estimates in other urban slums in Kenya are lacking. However, two studies performed in rural western Kenya also found high *Shigella* isolation rates of 15% and 16% of stool samples collected from patients presenting with diarrhea or dysentery [30], [31]. While we documented high rates of shigellosis in an urban slum, we do not have conclusive evidence indicating that rates were higher than in rural areas. However, high population density coupled with grossly inadequate sanitary facilities and unregulated water connections with high likelihood of contamination may contribute to the high incidence of shigellosis in this urban slum site.

As expected, patients presenting with dysentery were more likely to have *Shigella* isolated from stool than patients presenting with non-dysenteric diarrhea [30]. Like in other studies [3], [32], [33], [34], we found relatively low incidence of shigellosis among infants. This may be partially explained by passive induced immunity conferred by breastfeeding, as seen in a study conducted in Dhaka, Bangladesh [34], but may also result from fewer opportunities for exposure to contaminated food or water sources, especially when compared with toddlers. According to the Kenya demographic and health survey, 2008–2009, the mean duration of breastfeeding among Kenyan children is 21 months. It is possible that majority of mothers in our setting breastfed for a period of at least 1 year. Our findings of lowest incidence of shigellosis in people ≥50 years old are also consistent with other studies; while unexplained, it may relate to acquisition of protective immunity or, perhaps, to safer hygiene and/or food preparation practices.

Females in the 35–49 year age group had a higher incidence of shigellosis, than males in the same age group. It is not clear why women in this age group were at increased risk of being infected with *Shigella*; however, it is conceivable that they had a greater risk of being infected by young children.

Our adjusted rates suggest that over 0.4% of people living in urban slum settings are stricken with shigellosis annually. Like other developing countries, Kenya is experiencing rapid urbanization (>3%/year) and migrants from rural to urban areas tend to settle in slums [35]. Extrapolating population-based incidence rates presented in this paper to the estimated 4.3 million Kenyans living in urban informal settlements similar to Kibera [25], we estimate that over 17,000 cases of shigellosis occur in Kenyan urban slums each year. Easy access to high quality health care and a relatively small surveillance population likely account for the occurrence of only one death related to shigellosis during the study period. However, given global estimates of shigellosis-associated case-fatality rates of 0.67% [3], it is reasonable to assume that approximately 117 deaths from *Shigella* may occur annually in Kenyan urban slums.

As in other developing countries *S. flexneri* was the most common species of *Shigella* identified in our study population [3]. While this information is important in guiding vaccine development, further subtyping is necessary to identify the most common serotypes.

*Shigella* was isolated throughout the year. This may be explained by the lack of adequate sanitary facilities and raw sewage flowing all year round in open drainage channels to which children are exposed potentially providing constant exposure to *Shigella*.

Many of the *Shigella* isolates were resistant to commonly available and affordable antibiotics, consistent with findings of previous studies from Africa [30], [36], [37], [38], [39]. Nearly all isolates were susceptible to ceftriaxone, nalidixic acid and ciprofloxacin. While current susceptibility to fluoroquinolone drugs is reassuring, rapid emergence of nalidixic acid and ciprofloxacin resistance elsewhere [10], highlights the importance of judicious use of these drugs to preserve their effectiveness for treating severe shigellosis and other life-threatening infections. Surveillance during the same study period in Kibera has shown emergence of nalidixic acid-resistant *Salmonella* strains [18], [40], which likely heralds decreased utility of fluoroquinolones for treating invasive salmonellosis [41].

While this study was unique in terms of its active surveillance for shigellosis and for providing reliable basis for rate adjustments, limitations may have led to underestimates or overestimates of the burden of disease. Because of transport requirements to the Kisumu laboratory, some stool specimens were processed >24 hours after collection. Cary-Blair solution preserves viability of *Shigella* [42]; however, ideally specimens should be processed within 6 hours of collection. Thus, there may have been loss of viable *Shigella* during shipping and storage.

In calculating, adjusted incidence rates, we assumed that persons with diarrhea who reported visiting any clinic had similar shigellosis incidence rates as those seen in the study field clinic. This assumption could be erroneous if there was a systematic bias in which persons with shigellosis tended to go to the designated study clinic at different rates than persons with other causes of diarrhea or dysentery. We believe that use of non-study field clinics occurred primarily because the study clinic was open only Monday-through-Friday during daylight hours; thus, we do not think that there would be intrinsic biases in the way we adjusted the incidence rates. We did not adjust the incidence rates based on the proportion of patients who met the case definition and did not provide a stool specimen. This is because a relatively small proportion of patients meeting our diarrhea case definitions provided stool specimen, which would have made further adjustments imprecise, potentially exaggerating the adjusted incidence rates. On the other hand, opting not to make this adjustment likely led to underestimation of incidence rates. In addition, patients with dysentery were more likely to provide a stool sample for culture and sensitivity studies compared to patients presenting with non-bloody diarrhea. This led to a higher adjustment factor among patients with diarrhea (without dysentery), which may over estimate the burden of shigellosis in this category of patients.

Drinking water, sanitation, and hygiene improvements within the rapidly multiplying and expanding informal settlements in Kenya, and elsewhere in Africa, are needed to reduce the risk of shigellosis and other enteric infections. Health education, including a focus on hand washing with soap, provision of safe drinking water and proper waste disposal are feasible strategies for containing the burden of shigellosis [43]. Given the slow progress of improving living conditions in informal settlements, these high incidence rates highlight the need to hasten the development of effective vaccines to control shigellosis in resource-limited settings.
