# Correction: Use of Population-based Surveillance to Define the High Incidence of Shigellosis in an Urban Slum in Nairobi, Kenya

**DOI:** 10.1371/journal.pone.0105031

**Published:** 2014-08-04

**Authors:** Henry N. Njuguna, Leonard Cosmas, John Williamson, Dhillon Nyachieo, Beatrice Olack, John B. Ochieng, Newton Wamola, Joseph O. Oundo, Daniel R. Feikin, Eric D. Mintz, Robert F. Breima

In the original article, we reported detection of 6 *Shigella dysenteriae type 1* (Sd1) isolates. Because of substantial interest in Sd1, formerly a common cause of epidemics of severe dysentery, we went back to the specimens and repeated the microbiology. We have confirmed that all *Shigella dysenteriae* isolated in our study were non-type 1. We have made the following corrections to the text:

- In the “Results” section of the Abstract, the first sentence should read, “*Shigella* species were isolated from 262 (24%) of 1,096 stool specimens.”
- The last sentence of the “*Shigella* isolation and incidence rates” section of the Results should read, “For the period; 1 May, 2008 through 31 Dec, 2010, 242 (23%) Shigella bacteria were isolated from 1,096 stool specimens (data not shown).”
- The “Species distribution” section of the Results should read, “Most *Shigella* isolates were *S. flexneri* (64%) followed by *S. dysenteriae* (11%) *S. sonnei* (9%), and *S. boydii* (5%). Species could not be determined for 12% of isolates. All 27 isolates of S. *dysenteriae* were non-type 1 (Fig. 2).”
- In the last paragraph of the Results, “*Shigella* incidence and monthly rainfall” should be a section heading, and the subsequent text in that paragraph should be beneath it.

**Figure 2:**
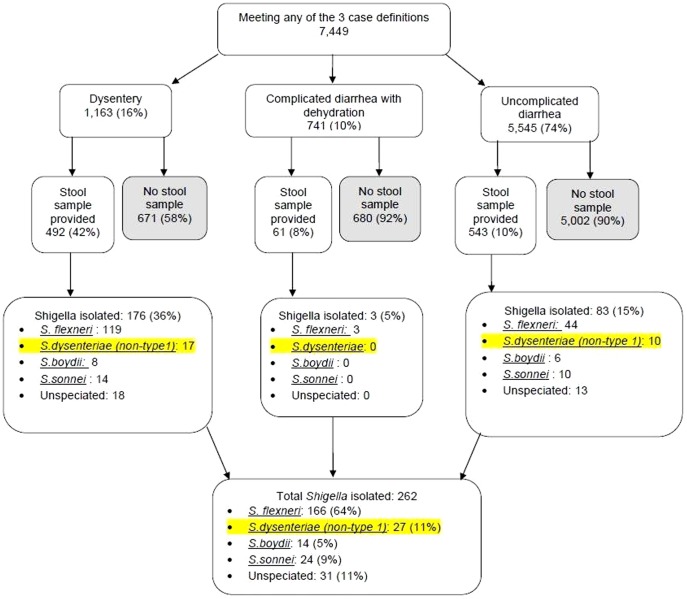
Flow chart illustrating distribution of diarrhea cases and shigella species isolated between 1 Jan 2007 and 31 Dec 2010 in Kibera, Kenya.

We have also revised Figure 2, which can be viewed here.
